# Mixed Methods Study Investigating Adolescent Acceptance and Implementation Outcomes of Serving Spicy Vegetables in School Lunch

**DOI:** 10.1016/j.cdnut.2024.104425

**Published:** 2024-07-25

**Authors:** Emily Siebert, Soo-Yeun Lee, Carter Philips, Melissa Pflugh Prescott

**Affiliations:** 1 Division of Nutritional Science and Food Science and Human Nutrition, University of Illinois, Urbana-Champaign, IL, United States; 2 School of Food Science, Washington State University, Pullman, Washington, DC, United States; 3 Department of Nutrition, Case Western Reserve University School of Medicine, Cleveland, OH, United States

**Keywords:** adolescents, school nutrition, implementation science, cultural humility, chili pepper

## Abstract

**Background:**

Only a few adolescents are meeting their daily vegetable requirement. At the same time, spicy food is increasingly popular and familiar across cultures.

**Objectives:**

To explore the implementation of spicy vegetables into school meals, the primary objective is to determine adolescents’ preferred degree of hot spice on steamed broccoli. Secondary objectives include estimating the appropriateness and acceptability of spicy vegetables in the National School Lunch Program and identifying strategies to promote spicy vegetables within school meals.

**Methods:**

One hundred participants between the age 11 and 17 y sampled 4 steamed broccoli florets with varying levels of a ground red and cayenne pepper spice blend (0, 0.9, 2.0, and 4.0 g). Participants rated their likeability of each broccoli sample on a 9-point hedonic scale and answered a survey assessing chili liking, chili consumption patterns, appropriateness, and acceptability. An interview assessed perspectives on spicy vegetables within school lunch.

Regression analyses assessed relationships between participant attributes and sample ratings and survey outcomes. Agglomerative hierarchical cluster analysis was conducted to cluster together participants with similar sample liking ratings.

**Results:**

Seventy-seven percent of participants reported that chili pepper makes food taste better, and 67% consumed spicy food weekly or daily. Chili likers (*n* = 41) were the dominant cluster group, compared with moderates (*n* = 31) and chili dislikers (*n* = 28). Thematic analysis results suggested that most participants support incorporating spicy vegetables into school lunch but mushy vegetable texture may undermine the impact of changing school vegetable spice levels.

**Conclusions:**

Spicy foods are commonly consumed by adolescents, and these findings support the inclusion of spicy vegetables in school lunch. Additional research is needed to identify policies and practices to improve the texture of vegetables in school meals and determine additional strategies to support cultural humility in child nutrition programs.

## Introduction

Vegetables are important sources of many vitamins and minerals essential for growth and development [1], but unfortunately, only 2.0% of adolescents are meeting the Dietary Guidelines for Americans for vegetable consumption [2]. Although vegetable consumption remains low, spicy food has grown in popularity [3]. Globally, 1 in 4 people eat chilies daily [4]. Chili pepper is a part of the capsicum genus, and the actual spicy component perceived when ingested is called capsaicin [5]. Chili pepper consumption is recorded across several countries [6], and even eaten multiple times per day in Mexico [7] and Korea, for example. More specifically, Koreans report an average of 3.55 g/d consumption of chili pepper [8], which is equivalent to ∼2 teaspoons of cayenne pepper. Other Asian countries report an estimation of 2.5–8 g chili pepper per day [9], whereas Americans consume it less routinely, varying from daily or just a few times per week or never [6]. Given the worldwide popularity of hot peppers, this pungent flavor is likely a staple in the homes of American youth from a variety of cultures, but there is no evidence of widespread use of this flavor profile in school lunch vegetables. The National School Lunch Program (NSLP) serves >29.4 million students every day, and 71% of school lunches are free or for a reduced price [10], providing a key source of nutrition to children from low-income families who are at high risk of chronic disease and obesity [11,12].

Given the widespread use of spicy foods in non-White households, the absence of spicy foods in school meals would be incongruent with cultural humility, which is the ongoing practice and intention of honoring different backgrounds, beliefs, customs, and values with an emphasis on self-awareness [13]. Additionally, vegetables are the most wasted meal component in school lunches [14] and research has also demonstrated that adolescents prefer seasoned vegetables over plain [[15], [16], [17]], emphasizing the potential for culinary innovations within the NSLP to improve vegetable consumption.

Offering spicy food in school lunch may offer additional health benefits as well. Capsaicin has been studied as a potential antiobesity, anticarcinogen, anti-inflammatory, and heart health component [[18], [19], [20]]. Acquiring a preference for spice can be increased through repeated exposure [6]. Increased exposure decreases the perceived burn over time, as those who have been consuming spicy food the longest have a higher tolerance [6]. Similarly, those who are frequently exposed to spicy food in childhood, have a higher preference for it in adulthood, emphasizing the importance of familiarity [21,22]. Given that the NSLP reaches millions of children every day and provides up to half of these student’s daily energy intake, the NSLP provides an opportunity to aid in the development of taste preferences for spicy foods among American youth and use spicy vegetables as a mechanism to improve vegetable consumption. To integrate spicy vegetables in school lunch it is important to understand what degree of spice will be most accepted and tolerated, as research concludes that spicy food liking is determined by many factors [6].

Little is known about adolescent spicy food preferences, although spicy snacks are widely available throughout vending machines and American grocery stores and are virally praised in songs targeted to kids [23]. In addition, the integration of spicy foods into school meals has not been studied with an implementation science lens. Implementation science is integral to the adoption and sustainment of research evidence in everyday use. Implementation outcomes, such as intervention acceptability and appropriateness, inform researchers and policymakers of the utility of new interventions, which can prevent investment in interventions that are unlikely to succeed in the real world [24]. Thus, the assessment of youth perspectives on whether spicy vegetables would be enjoyed and welcomed in school lunch and how best to introduce and promote them in schools are critical to understanding the potential utility of this intervention and developing appropriate implementation strategies. This study aims to address these research gaps with 3 objectives: The first is to identify the preferred spice level of steamed broccoli among adolescents. The second is to estimate the appropriateness and acceptability of spicy vegetables in the NSLP, and the third is to identify strategies to announce and promote spicy vegetables in school meals

## Methods

This convergent parallel mixed method study [25] consists of concurrently collected qualitative and quantitative data. Recruitment goals were set at 100 participants, which is a standard and sufficient sample size for sensory tests [26,27]. Recruitment took place from July to December 2022 and consisted of 2 recruitment mechanisms. First, parents of potential participants were recruited through a weekly e-mail newsletter sent to University of Illinois faculty and staff promoting campus announcements and events. Second, participants and their parents were recruited indirectly by emails and phone calls to local Boy and Girl Scout troops, one private school (which participates in the NSLP), a local school district, and the local chapter of the Boys and Girls Club of America to distribute flyers to announce the opportunity to participate in the study. Before data collection, communication was primarily between researchers and parents, but some older participants contacted the research team with questions about the study. Participants were required to be between the age of 11 and 17 y and be willing to sample broccoli with hot spices to participate in the study. These criteria and parental consent were assessed via a Qualtrics webform completed before data collection, and consent was confirmed, in person, on the day of data collection. Participants signed written assent forms and confirmed their willingness to try spicy foods in person on the day of data collection. Each participant received $15 compensation for participation. The experimental protocol was reviewed and approved by the Institutional Review Board, IRB # 23069.

### Ingredients and sample preparation

Broccoli was chosen as the vegetable for the consumer preference taste test based on evidence that suggests that dark green vegetables are the least consumed among other vegetable groups in school lunch [28]. It retains spice well, and seasoning broccoli has been shown to increase liking scores [29]. Four samples of steamed broccoli representing increasing levels of spice, with one sample representing the control (with no hot spice) were utilized in the sensory portion of the study to help identify the preferred spice level. Chefs provided input on the amount and type of spices used and researchers held taste tests and recipe trials with culturally diverse adult volunteers. Broccoli florets were purchased through Gordon Foods and kept frozen until use. All broccoli was cooked in a commercial kitchen facility. Each broccoli sample batch was made with 1 lb frozen broccoli, 9.7 g of canola oil, the predetermined hot spice mix of ground cayenne and red pepper and salt, or just salt for the control sample. The control contained 0 g of ground cayenne and red pepper. The low spice sample contained 0.6 g of ground pepper and 0.3 g of ground cayenne pepper. The medium spice sample contained 1.5 g of ground red pepper and 0.5 g of ground cayenne pepper, and the high spice sample contained 3.0 g of ground red pepper and 1.0 g of ground cayenne pepper. All 4 samples were mixed with 0.6 g of iodized salt. Two 1 lb batches of broccoli were steamed in 2 perforated pans in a salamander for 4 min at a time for the first 2 samples, and the 2 subsequent batches were weighed and steamed after participants finished the first sample. Once cooked, steamed broccoli was tossed with oil and preweighed spice and salt seasonings. One sample was served at a time, whereas the other sample was held in a warmer buffet tray.

### General protocol

**FIGURE 1:**
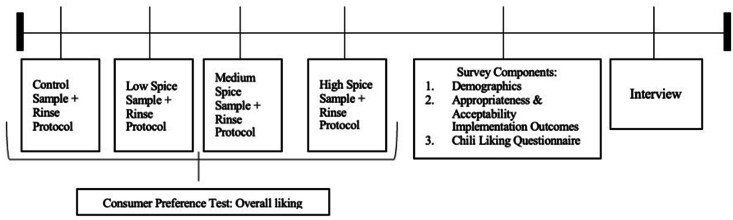
Data collection timeline.

Consented participants were scheduled for a 1-h research time slot. The timeline of data collection is depicted in Figure 1. Participants were asked to refrain from eating spicy food 24 h before and eating any other food items 30 min before their scheduled appointment. Quantitative and qualitative data collection occurred in a restaurant setting on a university campus when the restaurant was not in operation. Tables and chairs were set up in a restaurant style, with multiple chairs and tables under incandescent lighting. Soft music was played in the background to encumber participants from hearing each other. Participants were seated at separate tables in chairs facing away from one another to prevent verbal and nonverbal communication interactions. Each 1-h time slot had anywhere from 1 to 14 participants. The research team gave verbal directions to the protocol group before the consumer preference test, explaining the hedonic scale and rinse protocol, and participants were asked not to discuss their sensory ratings with anyone else in the room. As for sample presentation, each participant was served a tray that included a broccoli floret in a 2 oz cup labeled with a designated 3-digit code, room-temperature water, 1 empty spit cup, 2 oz of vanilla ice cream, and a napkin and spoon for the rinse protocol. Dairy is often used in sensory tests with spicy food because dairy contains a protein that helps break down capsaicin [30]. Ice cream was chosen as the dairy component to cater to the participant age group and reduce burn crossover from sample to sample. For the rinse protocol, participants were asked to expectorate the water and ice cream rinse to avoid carryover effects and to ensure consistent tasting intensities [31]. Verbal and written instructions directed the participants to rate their overall acceptance and liking on a 9-point hedonic scale with 1 = “dislike extremely” and 9 = “like extremely.” A member of the research team was always available to address any questions and supervise the participants.

### Survey measures for appropriateness and acceptability of spicy vegetables in school lunch

To address objective 2, which was to estimate the appropriateness and acceptability of spicy vegetables in school meals, a multicomponent survey was distributed to all participants after the sensory evaluation. This survey assessed demographics, including gender identity, questions about their spicy food preferences using a validated chili-liking questionnaire [32], and a validated survey assessing the student’s perspectives on the appropriateness and acceptability of including spicy broccoli in school lunch with agree/disagree statements. These validated acceptability and appropriateness implementation measures have been used in various research and technical settings to help predict implementation success of an intervention strategy [33,34]. The appropriateness and acceptability survey each contained 4 items for a total of 8 questions at the fifth-grade reading level and were adapted with “spicy broccoli” as the newly implemented intervention [33,34].

### Interviews

The following and final portion of the study was an open-ended interview in the dining room conducted by trained student research assistants and the senior author, a seasoned qualitative researcher. None of the participants had established relationships with the researchers. The interview included 8 questions pertaining to students’ thoughts on spicy vegetables in school lunch (including questions relating to the appropriateness and acceptability of spicy broccoli), with an optional 14 probing questions if participants struggled to answer the primary questions. These questions were designed to provide further insight on the second research objective and directly answer the third objective. The interview questions were primarily designed to assess objective 2 and provide key context to objective 1, allowing participants to elaborate on any opinions not shared in the survey and sensory study component. Participants were also asked to share promotional strategies to support spicy vegetables in school lunch to address objective 3. Interviews were audiotaped, transcribed verbatim, and reviewed for accuracy by 3 undergraduate research assistants.

### Statistical analysis

The variable chili liking was established based on the chili-liking survey’s true false statement, “I think chili liking makes food taste better.” Statistical analyses were performed using R version 4.2.2 (2022-10-31, R-project) [35] and XL-STAT (Addinsoft) [36]. Linear regression analysis was used to assess which participant attributes significantly predicted sample ratings. Participant attributes of interest included gender, age, age group (middle or high school), race, language, chili liking, and frequency of chili consumption. Logistic regressions were performed to assess which demographic variables significantly predicted chili liking. The 6 participants in the gender nonconforming category were excluded from the regression analysis as there was insufficient sample variation. Agglomerative hierarchical cluster (AHC) analysis was conducted using XL-STAT to cluster participants with similar liking ratings for the 4 samples. Once clusters were identified, sample liking data for all 4 samples were analyzed by analysis of variance (ANOVA) to determine if sample ratings significantly differed within clusters. When significance was identified, Fisher’s least significant difference test (LSD) was conducted with a 95% confidence level for pairwise comparisons test on sample liking from the sensory test data to assess how the samples are significantly different from each other. Additionally, Chi-square tests were used to evaluate differences between demographic variables across clusters. The significance level was set at 0.05. When missing data occurred, complete cases were used.

### Qualitative analysis of interview data

Qualitative analysis of open-ended interview data was performed using ATLAS.ti [37] (Version 22). Using a hybrid deductive and inductive approach, all 100 interviews were independently coded by 2 researchers based on a codebook with 14 primary codes with 0–26 supporting subcodes. The 2 researchers then compared their independent codes, and the data were coded according to their consensus. A third researcher resolved any coding discrepancies. Although data saturation was achieved midway through the data analysis process, all collected interviews were analyzed to make use of all existing data and provide research experiences for the student research assistants. Interview transcripts were not returned to participants for comment.

## Results

All participants (*n* = 100) completed the consumer preference sensory test, survey, and interview, however, not all participants answered every question within the survey. One hundred participants completed the acceptability portion of the survey, 99 participants completed the appropriateness portion of the survey, and 95 participants completed the chili-liking questionnaire.

Participants (*n* = 100) had a mean age of 13.02 ± 1.75 y, ranging from 11 to 17, with 48% female, 46% male, and 6% gender nonconforming, which comprised the combined categories of nonbinary, prefer not to say, or transgender. Forty-eight percent of participants reported White race, 28% reported Black or African American, 12% reported Asian race, and 11% reported Biracial, meaning they marked >1 race. Participant demographics are listed in Table 1. Out of 96 participants who answered the chili liking true and false statement, 74% (*n* = 71) marked “True” for “I think chili pepper makes food taste better,” highlighting their overall preference for spicy food or foods containing chili pepper. Out of the 99 participants that answered the question “How frequently do you eat all types of spicy foods, including, Mexican, Indian, Chinese, and other foods that contain chili pepper and cause tingling or burning?,” 20 participants (20.2%) reported consumption at least once per day, 30 participants (30.3%) reported consuming spicy food 3–4 times a week, and 21 participants (21.2%) reported consuming spicy food 1–3 times per month. Differences in the frequency of spicy food consumption by gender, age, race, and fluent languages spoken and demographics for chili liking based on questionnaire responses are reported in the Supplemental File as Supplemental Tables 1 and 2. Logistic regression results showed that chili liking did not significantly vary across sample demographics. Those that marked “True” to “Do you think chili pepper makes food taste better” included a variety of all races, ages, and genders.

**TABLE 1:** Demographics of participants (*n* = 100)

Demographics		
	Mean	SD
Age (y)	13.02	1.74
Gender identity		
	*N*	%
Female	48	48
Male	46	46
Gender nonconforming	6	6
Ethnicity		
Hispanic/Latino	5	5
Non-Hispanic/non-Latino	82	82
Not reported	13	13
Race		
Asian	12	12
Black or African American	28	28
White	48	48
Biracial	11	11
Not reported	1	1
Language		
English only	85	85
Multilingual	15	15

There were 4 key themes that emerged from the qualitative interview findings. As shown in Table 2 these 4 themes are: The Potential For Hot Spices to Improve Vegetable Intake, Varying Preferences for Spicy Food, Poor Perceptions of School Lunch Vegetables, and spicy food popularity. These findings were integrated into the results for research objectives 1 and 2.

**TABLE 2:** Key interview themes and supporting illustrative quotes (*n* = 100)

Themes	Quotes
Potential for hot spices to improve vegetable intake	“I think [spicy vegetables] would be better for kids because most kids don’t eat their vegetables” “I think [spicy vegetables] are a great idea because I feel like kids now like spicy foods a lot, so I feel they would eat vegetables more” “I think most kids at my school would just eat [spicy vegetables] regardless, because everyone at our school likes spicy things”
Varying preferences for spicy food	“I think that for me personally, I do have a higher spice tolerance than others so I would enjoy it. I'm not quite sure about other people” “Sometimes [spicy food] can be too much for people.” “Different people have different spicy preferences.”
Poor perceptions of school lunch vegetables	“I think of [the vegetables at school] as poor quality and usually, when I’ve seen them in the past, they come off as usually overcooked.” “They look like they’ve been sitting in a jar for a while, smushed.” “[School lunch vegetables are] all raw, and they don’t taste very good. People touch it with their hands and stuff. I don’t know. I don’t really like it.”
Spicy food popularity	“I think [spicy vegetables] are very fitting, because a lot of the kids in school like spicy things. There’s a lot of kids who just bring spicy snacks and stuff to school, so I think it’s fitting for the school cafeteria.” “I think [students/peers] would like spicy food in general. Really don't matter. Just put [spices] on [school lunch foods]. If it's spicy it'll catch their eye. Just any spicy food.”

### Preferred spice level of steamed broccoli among adolescents

**FIGURE 2:**
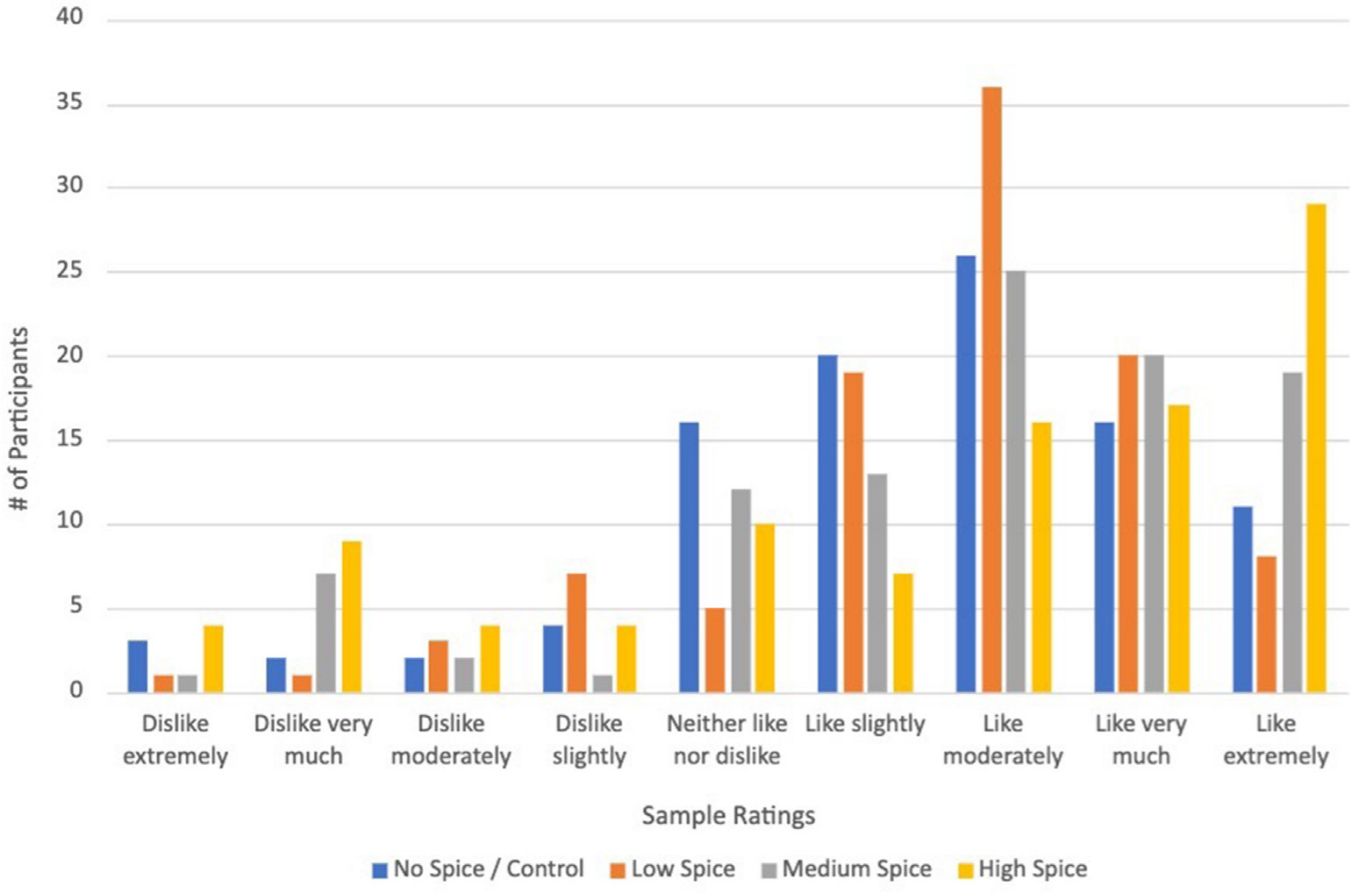
Hedonic ratings for 4 broccoli samples.

All samples were rated as “like slightly” on the hedonic scale, with samples ranging from the control to high spice averaging 6.4 ± 1.8, 6.6 ± 1.6, 6.7 ± 2.0, 6.5 ± 2.5, respectively. There were no significant differences in ratings across the sample (*P* = 0.6), and the range for every sample was 1–9, illustrating diverse taste preferences across all participants. The mode for the high spice sample was 9, representing “like extremely,” whereas the mode for the other samples was all 7 representing “like moderately.” The rating distribution for all 4 samples is provided in Figure 2. As expected, the linear regression revealed that chili liking was significantly associated with ratings for the spicy broccoli samples. As shown in Table 3, chili likers rated the high spice sample 2.65 points higher than those who indicated dislike of chili on the survey. Race, ethnicity, language, age, age group (middle compared with high school), and frequency of spicy food consumption were not included in this linear regression because they were not significant predictors of spicy broccoli liking.

**TABLE 3:** Linear regression analyses examining significant predictors for sample ratings

Explanatory variable	Sample 2	Sample 3	Sample 4
	β (SE)	β (SE)	β (SE)
Chili Liking (true)	0.92 (0.41)∗	2.03 (0.48)∗∗∗	2.65 (0.57)∗∗∗
Gender (male)	0.09 (0.33)	0.71 (0.39)	0.93 (0.47)

Linear regression analyses were used for spicy broccoli sample rating outcomes, “**.**” 0.1 ∗ *P* < 0.05 ∗∗ *P* < 0.01, ∗∗∗ *P* < 0.001. Race, ethnicity, language, age, age group (middle vs. high school), and frequency of spicy food consumption were not included in this linear regression, because they were not significant predictors of spicy broccoli liking.

**FIGURE 3:**
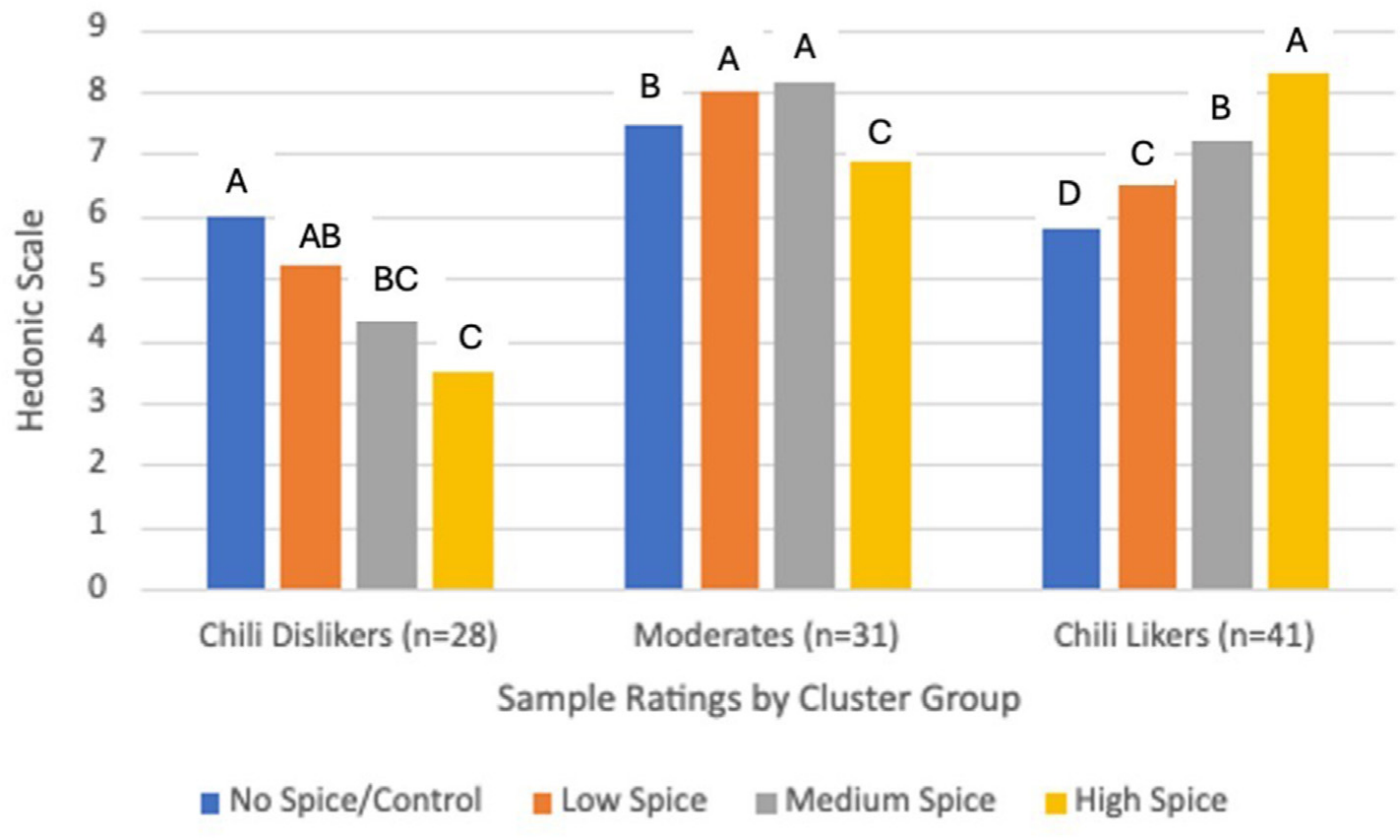
Overall liking of each sample by cluster group. The samples with the same letters within each cluster are not significantly different (*P* < 0.05) from each other.

AHC cluster analysis revealed 3 distinct clusters from participant liking scores across all 4 samples. ANOVA within each cluster revealed that there is a significant difference across sample ratings that shows different patterns of liking within each cluster (*P* < 0.0001 for all 3 clusters). Hedonic rating trends for each sample by cluster groups are depicted in Figure 3. The chili dislikers cluster (*n* = 28) consisted of participants who disliked the spicy broccoli; Figure 3 illustrates their downward liking trend as the broccoli samples increased in heat. The moderates cluster (*n* = 31) represented participants that favored the midrange spice levels (low spice and medium spice samples). The chili likers cluster (*n* = 48) consisted of participants who rated the spicy samples the highest (medium spice and high spice samples), as sample acceptance ratings significantly increased when spice levels increased. The means for each cluster are depicted in Table 4. Chili likers rated the high spice sample the highest with a rating mean of 8.3 compared with the chili dislikers who rated the high spice sample the lowest with a mean of 3.5. The moderates positively rated the low and medium spice samples and the control sample higher than the high spice sample. Furthermore, as shown in Figure 3, Fisher’s LSD revealed that the control sample ratings were significantly different from the high spice sample ratings, and only the chili likers had significantly different liking ratings across all 4 samples. The specific pairwise sample comparisons are reported in the Supplemental File as Supplemental Table 3. When comparing participant attributes between each cluster using Chi-square analysis, shown in Table 5, the chili likers cluster were primarily male, and the chili dislikers were primarily female. Gender identity was significantly different across clusters (*P* = 0.02) as well as chili liking (*P* = 0.003).

**TABLE 4:** Means for overall liking by clusters

Cluster	Steamed broccoli samples			
	Control	Low spice	Medium spice	High spice
Chili dislikers (*n* = 28)	6.0	5.2	4.3	3.5
Moderates (*n* = 31)	7.5	8.0	8.1	6.9
Chili likers (*n* = 41)	5.8	6.6	7.2	8.3

On a 9-point hedonic scale, anchored 9 = like extremely, 1 = dislike extremely.

**TABLE 5:** Participant attribute breakdown by cluster (%)

	*N* = 100	Participant attributes by cluster (%)		
		Chili dislikers *n* = 28	Moderates *n* = 31	Chili likers *n* = 41
Gender identity∗	Male	28.5	51.6	56.1
	Female	67.9	41.9	36.6
	Gender Nonconforming	3.6	6.5	7.3
Age (y)	11–13	82.1	54.8	68.3
	14–17	17.9	38.7	29.3
	Not reported	0	6.5	2.4
Race	Asian	14.3	16.1	7.3
	Biracial	7.1	22.6	4.9
	Black	25	22.6	31.7
	White	53.6	38.7	53.7
	Not reported	0	0	2.4
Language	English	85.7	77.4	90.2
	Multilingual	14.3	22.6	9.8
Ethnicity	Hispanic/Latino	7.1	0	7.3
	Non-Hispanic/Latino	71.4	87.1	85.4
	Not reported	21.4	12.9	7.3
Spicy food consumption frequency	Monthly–yearly	46.4	25.8	26.8
	Weekly	35.7	54.8	48.8
	Daily	17.9	16.1	24.4
	Not reported	0	3.2	0
Chili liking∗∗	True	53.6	87.1	85.4
	False	39.3	9.7	12.2
	Not reported	7.1	3.2	2.4

Chi-square analysis assessing gender differences by cluster excluded gender nonconforming participants from the analysis. Chili liking is based on the question: “I think chili makes food taste better? T/F” ∗*P* < 0.05 ∗∗*P* < 0.01.

As observed in theme 1, spicy food popularity, qualitative findings are generally consistent with these quantitative findings. Spicy food in general was recognized as liked and often consumed among students. Participants mentioned things like “spicy food is so great and so tasteful” and “lots of kids like spicy foods, especially at my school.” Hot chips like Hot Cheetos and Takis were brought up by a group of participants as well, stating “[other kids] like spicy food, they like Hot Cheetos,” illustrating the general acceptance of spicy flavors within this age group.

## Appropriateness and acceptability of spicy broccoli in the NSLP

**FIGURE 4:**
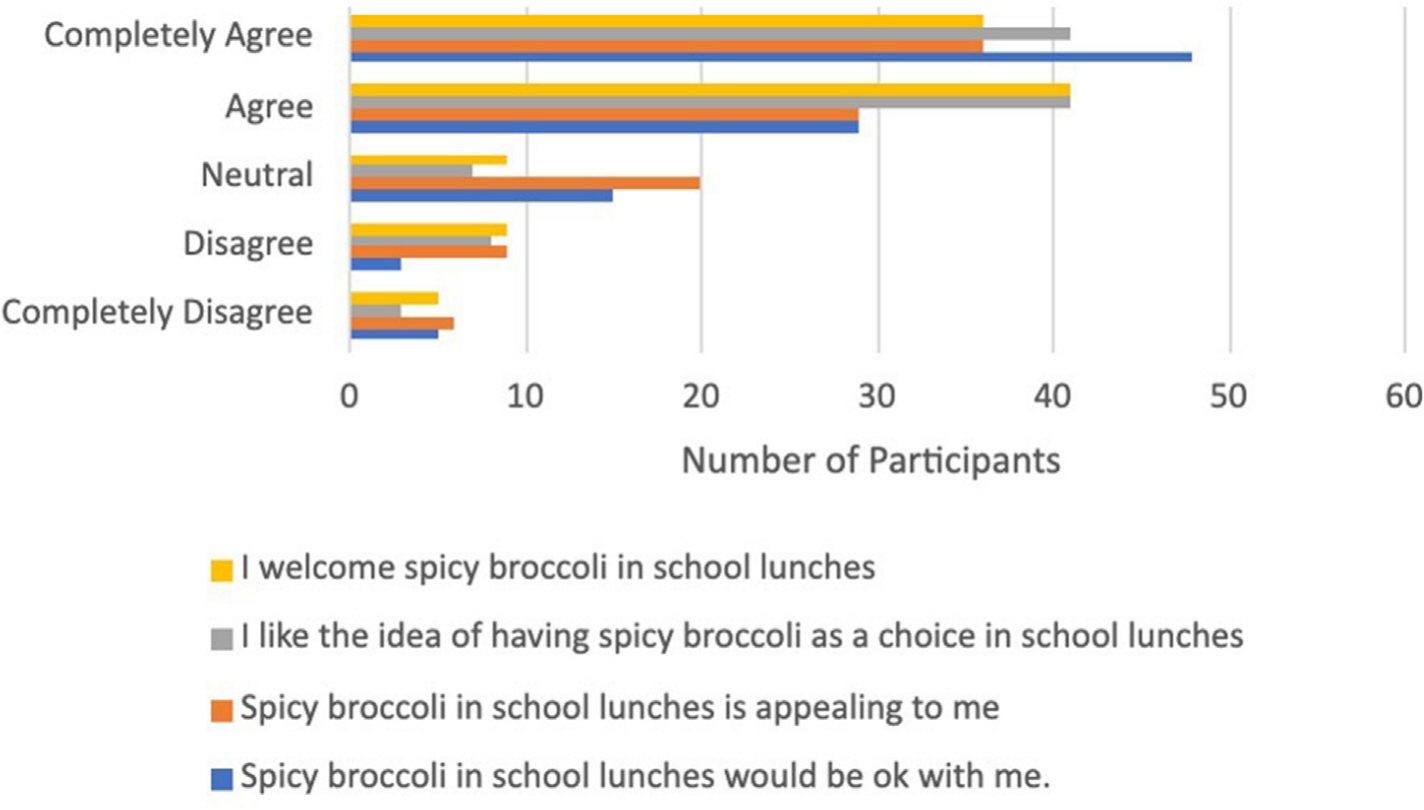
Survey results for acceptability ratings for spicy broccoli.

**FIGURE 5:**
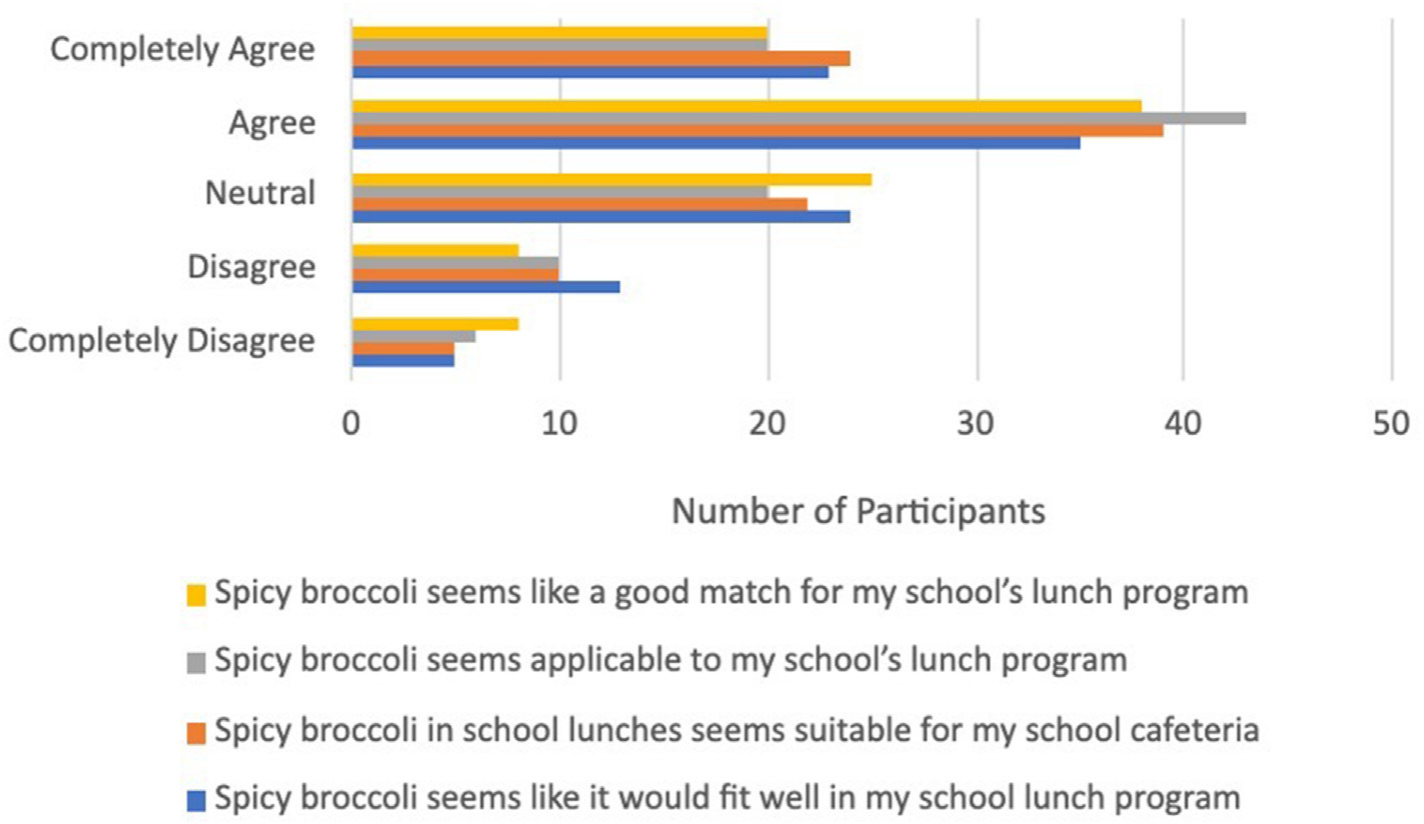
Survey results for appropriateness ratings for spicy broccoli.

As shown in FIGURE 4, FIGURE 5, respectively, participants rated the acceptability (mean 4.0 ± 1.0) of spicy broccoli in school lunch slightly higher than the appropriateness (mean 3.6 ± 1.0). According to participant interview data, the 3%–6% who “completely disagree” with the overall acceptability of spicy broccoli in school lunch were either chili dislikers, did not like broccoli, or expressed concerns for varying spice tolerances and preferences among students. The latter was demonstrated by the theme Varying Preferences for Spicy Food. Students mentioned, “some people would like it, some people won’t,” and “I don’t know if other people in my school would like it.” Nineteen participants raised a concern for introducing spicy vegetables, and 14 participants recognized the diversity of spice level preferences and tolerances. Many participants suggested a solution to this issue: “I think having both a spicy and nonspicy option would be good as well for some people whose spice tolerance isn’t that high.”

Those who marked “completely agree” with the overall acceptability of spicy broccoli rated the spicy broccoli samples higher relative to those who did not “completely agree” with the appropriateness and acceptability statements. As for appropriateness, the 5%–8% selecting “completely disagree” with the overall appropriateness of spicy broccoli in school lunch were comprised of a mix of chili likers and dislikers, and a majority expressed concern in the interview regarding varying spicy food preferences, or the schools’ unappealing and mushy vegetable offerings. Qualitative data supporting the theme of Poor Perceptions of School Lunch Vegetables suggest that these concerns are driven by poor opinions of the school’s cooking and serving methods for vegetables and can be characterized by 2 types of complaints: poor taste and unpleasant texture. The taste of vegetables served at school was commonly described as “horrible,” “sad,” “gross,” “nasty,” “bland.” The texture was described as “soggy,” “squishy,” “mushy,” “smushed.” A small group of participants described the vegetable offerings as unsanitary as well, saying “people touch it with their hands and stuff,” but this may refer to school salad bars specifically.

The final theme of Potential for Hot Spices to Increase Vegetable Intake further demonstrates the appropriateness of integrating spicy vegetables into school meals. Spicy vegetables were generally viewed positively or as a good idea by 75 participants. Seventy-seven participants said they would put spicy vegetables on their trays if spicy vegetables were offered in the lunch line. Students perceived spicy vegetables as “more flavorful,” “a nice addition,” “a good option,” “good for kids to eat more vegetables,” and believed “people would eat them.” One student added, “I think more people would eat [vegetables] if it was spicier.” There was some overlap between this theme and spicy food popularity, as spicy vegetables were acknowledged as something that might be popular given the popularity of spicy food in general. A student said: “If (students) saw spicy vegetables in the lunch line... because some kids actually like spicy stuff, and so if they knew that we had spicy vegetables, then I think they’ll actually try to get them.”

### Strategies to announce and promote spicy vegetables in school lunch

Participants provided 24 different promotional methods for spicy vegetables in their schools. The most recommended strategies included posters, banners or signs, and school announcements. One participant suggested, “maybe put posters in front of the cafeteria and be like ‘New spicy food’ or something like that.” Another recognized, “usually when there’s something new in the cafeteria, there’s something advertising it somewhere, so then a lot of people get it, even if they don’t usually get stuff like that. They’re like “Ok, it’s new. I’m going to try that.” Other popular ideas included a trial run with spicy vegetables, incentives for trying spicy vegetables, posting on social media, and word of mouth.

## Discussion

Adolescent hot spice preferences and the inclusion of spicy additives in school lunch have been minimally studied. The majority of participants felt that chili pepper makes food taste better, and 67% of the participants consumed spicy food at least weekly, as measured by the validated chili-liking survey. Although the sample ratings did not significantly differ overall, all samples were generally rated as liked, and chili likers comprised the largest cluster size. These quantitative findings are consistent with those of the interviews, which found most students feel that serving spicy vegetables in school lunch is worthwhile and most would select spicy vegetables in their own school lunch. Students desire more options and suggest a spicy and nonspicy vegetable choice. Participants’ suggestions could be honored by provision of spicy vegetables in the hot lunch line, whereas salad bars could provide a nonspicy alternative. This choice would cater to various preferences and facilitate cultural inclusiveness.

Gender significantly differed between clusters according to Chi-square analysis. This is consistent with outside literature, which states that males have a higher preferred degree of spiciness compared with females [[38], [39], [40]]. This may be attributed to testosterone [41], gender personality differences [42], or social masculinity standards [7]. More males than females participate in the NSLP at all grade levels altogether, further suggesting the appropriateness of integrating spicy vegetables into middle and high school nutrition programs [43]. NSLP participation rates also vary by race, with an estimated 66.2% of Black non-Hispanics, 64.4% of Hispanics, 42.7% of White non-Hispanic students, and 30.9% of non-Hispanic Asian participating in the program in 2021 [44]. Chili pepper has been recognized as a central component within Hispanic and Latinx culture and cooking [7], further supporting the cultural appropriateness of spicy vegetables in school lunch. People of color’s diets are often disparaged as unhealthy [45], but chili peppers are a dietary component that can provide various health benefits such as antioxidants and weight management potential [46]. Adding this culturally familiar spice allows people of less-dominant cultures to be better included in school nutrition programs. Interestingly though, race, ethnicity, or language did not predict sample ratings and did not significantly differ between clusters, supporting the notion that taste preferences and eating habits should not be stereotyped by race- and culture-related demographic factors. In the interviews, participants also commented on their willingness to try others’ cultural foods, further demonstrating how hot spices can rectify flavor, menu variety, and cultural inclusivity.

Although our mixed methods findings suggest the potential for spicy foods to improve school lunch vegetable consumption, they also suggest that the addition of hot spice alone may not be sufficient to change student dietary behavior. The control broccoli sample with just oil and salt was rated as liked overall, but the interview findings showcased that vegetables at school commonly have an unpleasant texture. Mushy vegetables at school have been recognized in other literature as well. For example, in a school plate waste evaluation study, Zhao et al. [47] found that improved quality of vegetables may reduce food waste as students believed that it was acceptable to throw away disliked food and described the food as “prepared in a clumpy and soggy way.” Altogether, this suggests that texture and sensory appeal contribute to low vegetable consumption at school and emphasizes the need for thoughtful vegetable preparation efforts in school cafeterias. In another mixed methods study investigating fruit and vegetable intake in schools, Hamdi et al. [48] encountered low-quality broccoli during lunch service. The broccoli served at lunch was prepared before lunch service and held throughout multiple lunch periods, resulting in visibly poor broccoli texture, suggesting the need for batch cooking and other strategies to avoid overcooking vegetables.

Other researchers investigated strategies to make vegetables more palatable. For example, in a randomized controlled trial a chef worked to improve school meal palatability. The vegetable selection increased in schools with a chef compared with the control schools [49], showcasing the importance of vegetable taste and appeal. Hiring a chef is likely not feasible for all schools across America, but these findings further elaborate the need for increased focus on how vegetables in lunch are prepared. The addition of spices and herbs to vegetables in the NSLP has been previously studied and found to be successful in increasing vegetable intake [50]. This is consistent with the qualitative and quantitative results of the present study, which found that participants generally viewed spicy vegetables as an acceptable and appropriate addition to school lunch through surveys and interviews. Taste is the dominant factor in food choice, especially among children [51], and adolescence is a critical period for forming healthy eating habits [52]. This emphasizes both the importance of serving healthy food that students believe will taste good and the public health significance of serving quality vegetables in schools.

### Strengths and limitations

The present study findings should be considered alongside its limitations. Sensory tests are extremely sensitive and hold potential for various biases and limitations [53]. Logical error occurs when panelists make assumptions about the sample. This error possibly occurred for the low-to-high spice samples because the ground red pepper and cayenne resulted in red speckles on the spicy broccoli samples but not on the control; therefore, participants may have expected a spicy flavor based on this visual cue. Even though this may have influenced their hedonic perceptions, this sensory cue of color would be visible in the lunch line, which is an essential consideration for selection behaviors at school. It is also possible that expectation error occurred, which is the sensory bias that participants have preconceived expectations about the sample before trying and rating it. The samples were presented in order from the least spicy to spicy, and participants may have caught on to this order, which could influence their overall hedonic perception of the sample. One limitation that was not fully realized until after data collection is the lack of consistency between sample-tasting rinse protocols. The participants were instructed to expectorate the ice cream and water during the rinse but to swallow each broccoli sample. This is not a standard protocol in sensory evaluation, because there may be residual stimulation at the back of the tongue from swallowing the samples that may not have been completely cleansed with the rinse being expectorated. However, ice cream naturally melts when it hits the tongue, so it is likely that the ice cream reached the back end of the esophagus. Additionally, only one attribute of the broccoli sample was assessed as well: overall liking. The degree of consumer acceptability does not consider other perceptions or attributes such as taste, texture, visual appeal, however, more insights regarding the sample were reviewed in the interview. Another limitation of this mixed methods study is that no quantitative data were used to assess objective 3, which is somewhat unusual for the study design. However, using qualitative data alone did not appear to undermine the accuracy or clarity of the findings and provided a rich understanding of potential promotional strategies for spicy vegetables in school lunch. This study also lacked a quantitative assessment of general vegetable liking, and this would be expected to influence hedonic ratings of the broccoli sample, because a group of participants mentioned not liking broccoli in their interview. In similar research evaluating the effects of spices on vegetables, hedonic ratings differed by the 2 group’s vegetable preferences [29]. Lastly, this study did not measure participants’ participation in school lunch. However, the participation of all students, regardless of familial financial resources, in school lunch is advantageous to public health [54] and helps schools receive increased federal meal reimbursement. Subsequently, the authors maintain that the perspectives of all students attending schools that participate in the NSLP are important and relate to the potential success of serving spicy vegetables at lunch.

However, this mixed methods design holds several strengths as the quantitative and qualitative data results complemented each other and allowed for a richer assessment of the research objectives. The interview findings provided a general assessment of broccoli consumption and revealed more systemic problems within the school cafeteria such as mushy and low-quality vegetables. Our demographics are somewhat consistent with the current participation rates of the NSLP [44], however, they did not reflect Hispanic population well, with 64.4% of Hispanics participating in the NSLP, and 82% of our sample were non-Hispanic Latino. Given the relatively low participation of Hispanic youth in the study and the central role of chili peppers in many Hispanic foodways, this study may have been a conservative estimate of youth acceptance of spicy vegetables.

In conclusion, this research demonstrates the potential for incorporating chili pepper within school cafeterias to support vegetable consumption. Current policy focuses on nutrition standards and requirements but lacks solutions to taste and vegetable quality, demonstrating the need for more research and focus on these areas. Our study only addressed the taste aspect of vegetables, and to truly evaluate the impact of spicy vegetables on school lunch vegetable taste, future studies should determine how spices change the sensory attributes of vegetables prepared in schools, as our cooking method likely differed from school kitchens. Pungent spices alone may be an appropriate additive in school lunch to support other cultural foods and the diversity of NSLP participants, as spicy food popularity common in this age group. If not utilized on vegetables, pungent spices are an appropriate additive in school lunch to support other cultural foods and honor the diversity of NSLP participants. Overall, the spicy vegetable recipe used in this study is appropriate for translation to school nutrition programs due to its low ingredient costs and could also be adapted for other steamed vegetables. However, the perceptions of school nutrition staff on the inclusion of spicy vegetables in school meals are also critical to its implementation and should be assessed in the future. More research is also required to fully understand how spicy vegetables, alongside concurrent improvements in cooking and preparation methods, will impact school vegetable consumption and cultural inclusivity.

## Author contributions

The authors’ responsibilities were as follows – MPP: research conception; ERS, MPP, YSL, CP: research design and methodology and critical review of manuscript; ERS: data acquisition and analysis and draft of initial manuscript; ERS and MPP: full access to all the data in the study and take responsibility for the integrity of the data and accuracy of the data analysis; and all authors read and approved the final manuscript.

## Conflict of interest

The authors have no conflicts of interest or relevant financial interests to disclose.

## Data Availability

Data described in the manuscript, code book, and analytic code will be made available upon request pending application and approval.
