# Supplementary material for: Mixed Methods Study Investigating Adolescent Acceptance and Implementation Outcomes of Serving Spicy Vegetables in School Lunch
Source: Curr Dev Nutr. 2024 Jul 25;8(8):104425. doi: 10.1016/j.cdnut.2024.104425 (PMC11367512; doi:10.1016/j.cdnut.2024.104425)
Supplement: Multimedia component 1 [file mmc1.docx]

Supplemental File:

**Supplemental Table 1: Demographics by Frequency of Spicy Food Consumption** (n = 99)

|  | **Monthly- Yearly Consumption** | **Weekly Consumption** | **Everyday Consumption** |
| --- | --- | --- | --- |
| **Gender Identity** |  |  |  |
| **Male** | 11 | 24 | 10 |
| **Female** | 18 | 20 | 10 |
| **Gender Nonconforming** | 2 | 4 | 0 |
| **Age** |  |  |  |
| **Middle School (11-13)** | 23 | 32 | 13 |
| **High School (14-17)** | 8 | 14 | 7 |
| **Race** |  |  |  |
| **Asian** | 1 | 7 | 4 |
| **Black or African American** | 10 | 11 | 6 |
| **White** | 17 | 22 | 9 |
| **Biracial** | 3 | 7 | 1 |
| **Language** |  |  |  |
| **English Only** | 28 | 40 | 16 |
| **Multilingual** | 3 | 8 | 4 |

**Supplemental Table 2:** **Chili Liking by Demographics, “Do you think Chili Pepper Makes Food Taste Better”** (n=96)

|  | **TRUE** | **FALSE** |
| --- | --- | --- |
| **Gender Identity** | |  |
| **Male** | 38 | 6 |
| **Female** | 33 | 13 |
| **Gender Nonconforming** | 6 | 0 |
| **Age** | |  |
| **Middle School (11-13)** | 52 | 13 |
| **High School (14-17)** | 24 | 5 |
| **Race** | |  |
| **Asian** | 12 | 0 |
| **Biracial** | 9 | 2 |
| **Black or**  **African**  **American** | 20 | 6 |
| **White** | 35 | 11 |
| **Language** | |  |
| **English Only** | 64 | 18 |
| **Multilingual** | 13 | 1 |

## Supplemental Table 3: LSD Analysis of the Difference between Sample Rating Scores within Clusters

| **Cluster, LSD Value** | **Contrast** | **Difference** | **Standardized Difference** | **Critical Value** | **Pr > Diff** |
| --- | --- | --- | --- | --- | --- |
| Cluster 1, 0.9 | Sample 1 vs Sample 4 | 2.5 | 5.3 | 2.0 | **< 0.0001** |
|  | Sample 1 vs Sample 3 | 1.7 | 3.6 | 2.0 | **0.001** |
|  | Sample 1 vs Sample 2 | 0.8 | 1.7 | 2.0 | 0.099 |
|  | Sample 2 vs Sample 4 | 1.7 | 3.6 | 2.0 | **0** |
|  | Sample 2 vs Sample 3 | 0.9 | 1.9 | 2.0 | 0.061 |
|  | Sample 3 vs Sample 4 | 0.8 | 1.7 | 2.0 | 0.085 |
| Cluster 2, 0.5 | Sample 3 vs Sample 4 | 1.3 | 5.0 | 2.0 | **< 0.0001** |
|  | Sample 3 vs Sample 1 | 0.7 | 2.7 | 2.0 | **0.009** |
|  | Sample 3 vs Sample 2 | 0.1 | 0.5 | 2.0 | 0.61 |
|  | Sample 2 vs Sample 4 | 1.1 | 4.5 | 2.0 | **< 0.0001** |
|  | Sample 2 vs Sample 1 | 0.5 | 2.2 | 2.0 | **0.032** |
|  | Sample 1 vs Sample 4 | 0.6 | 2.3 | 2.0 | **0.024** |
| Cluster 3, 0.5 | Sample 4 vs Sample 1 | 2.5 | 9.8 | 2.0 | **< 0.0001** |
|  | Sample 4 vs Sample 2 | 1.7 | 6.8 | 2.0 | **< 0.0001** |
|  | Sample 4 vs Sample 3 | 1.1 | 4.2 | 2.0 | **< 0.0001** |
|  | Sample 3 vs Sample 1 | 1.4 | 5.6 | 2.0 | **< 0.0001** |
|  | Sample 3 vs Sample 2 | 0.7 | 2.6 | 2.0 | **0.011** |
|  | Sample 2 vs Sample 1 | 0.8 | 3 | 2.0 | **0.003** |

*Note: Fisher’s Least Significant Test analyses used to compare overall liking ratings between each sample within each cluster. Bolded values are significant*
